# ^18^F-fluorodeoxyglucose–positron emission tomography/computed tomography for the diagnosis of polymyalgia-like illnesses: a retrospective study

**DOI:** 10.1186/s41927-020-00121-y

**Published:** 2020-04-24

**Authors:** Hideyuki Horikoshi, Takashi Nakanishi, Katsumi Tamura, Fumihiko Kimura, Kenji Itoh

**Affiliations:** 1 Division of Hematology and Rheumatology, Department of Internal Medicine, National Defense Medical College, 3-2 Namiki, Tokorozawa, Saitama, 359-8513 Japan; 2 Tokorozawa PET Diagnostic Imaging Clinic, 7-5 Higashi Sumiyoshi, Tokorozawa, Saitama, 359-1124 Japan

**Keywords:** Polymyalgia-like illness, Polymyalgia rheumatica, FDG–PET/CT

## Abstract

**Background:**

Various inflammatory conditions may present with musculoskeletal symptoms similar to those of polymyalgia rheumatica (PMR). We investigated findings on ^18^F-fluorodexoxyglucose (FDG)-positron emission tomography/computed tomography (PET/CT) images that may differentiate PMR from polymyalgia-like illnesses.

**Methods:**

We analyzed data from 25 patients with new-onset polymyalgia-like illnesses who fulfilled Bird’s diagnostic criteria for PMR and had undergone FDG–PET/CT scan. To assess the uptake by major joints and synovial bursae, particularly at PMR-specific sites (shoulder, sternoclavicular, and hip joints, interspinous bursae, ischial tuberosities, and greater trochanters), we used visual scoring system to score FDG uptake: 0, no uptake (same as bone); 1, slight uptake; 2, moderate uptake (same as the liver); 3, greater uptake than the liver; and 4, uptake as strong as in the cerebellum.

**Results:**

The final diagnoses were PMR in 17 patients and non-PMR in eight patients (three malignancies, two infections, one cholesterol crystal embolism, one ANCA-associated vasculitis, and one undefined diagnosis). Although the serum MMP-3 levels were significantly higher in patients with PMR, C-reactive protein and erythrocyte sedimentation rate mean values did not differ between the two groups. In PMR-specific sites, FDG accumulations were observed in all cases of PMR, with a high PET-positive score of 2.00 (range, 0–3), but it was low in non-PMR cases, with a PET-positive score of 1.00 (range, 0–3).

**Conclusions:**

The FDG accumulation patterns in polymyalgia-like illness differ from those in PMR, despite the similar clinical presentations of both conditions. An FDG–PET/CT scan is useful for differentiating PMR from other polymyalgia-like illnesses.

## Background

Polymyalgia rheumatica (PMR) is a common inflammatory disorder that affects the elderly. It is characterized by muscle pain and stiffness in the neck, shoulders, and pelvic girdle [1]. However, the musculoskeletal symptoms associated with other inflammatory conditions such as infections, vasculitis, arthritis, myositis, and neoplasms may be similar and sometimes fulfill the diagnostic criteria for PMR [2, 3]. Therefore, it is often difficult to distinguish PMR from “polymyalgia-like illnesses” and their long differential diagnosis list [1, 4, 5].

The ^18^f-fluorodexoxyglucose (FDG)–positron emission tomography/computed tomography (PET/CT) imaging technique has been useful in the evaluation of joint inflammation [6, 7]. FDG accumulations in the shoulder and hip joints, interspinous bursae, and capsules of the knee are generally seen bilaterally on PET/CT images of patients with PMR [8–10], and FDG accumulations in the ischial tuberosities, greater trochanters, and lumbar interspinous bursae have also been reported [11]. The presence of uptake at two or more of these sites is reported to have high sensitivity (85.7%) and specificity (88.2%) for the diagnosis of PMR [11]. While FDG–PET/CT imaging in cases of polymyalgia-like illnesses such as paraneoplastic syndrome have also been reported [12, 13], no studies have compared the imaging findings between paraneoplastic polymyalgia (or other polymyalgia-like illnesses) and PMR.

## Methods

### Patients

We reviewed the clinical data of 25 patients with new-onset polymyalgia-like symptoms admitted to the Division of Rheumatology of our institution between 2008 and 2013. Seventeen (9 men, 8 women; median age 77 [60–89] years) were diagnosed as having PMR, and 8 (4 men, 4 women; median age 71 [50–81] years) were diagnosed as having diseases other than PMR (non-PMR). All patients with PMR and non-PMR met the PMR criteria of Bird et al. [14], and we also evaluated their clinical symptoms and blood test results of rheumatoid factor and anti-CCP antibody using the 2012 ACR/EULAR provisional classification criteria [15]. Blood samples were obtained during the first visit. Erythrocyte sedimentation rates (ESRs), and C-reactive protein (CRP) and MMP-3 levels were measured to evaluate the degree of inflammatory activity. MMP-3 concentrations were measured using latex-enhanced immunoturbidimetric assays (Kyowa Pharmachemical, Toyama, Japan; the upper limit of the normal reference range was 59.7 ng/mL for women, and 121.0 ng/mL for men). For further MMP-3 level comparisons, we collected serum samples from 31 untreated patients with RA having had the disease for less than 6 months. Additional Table S1 shows the basic characteristics of these patients. We analyzed laboratory and imaging findings retrospectively. Individual patients cannot be identified from the materials in the manuscript, and the review board of the National Defense Medical College approved the study protocol (reference number: 2595). We posted relevant study protocol information on the web site of our division, including the announcement for the patients about their right to read the study protocol and to refuse to be included in the study (http://www.ndmc.ac.jp/hospital/section/kogenbyo/).

### FDG–PET/CT imaging

The patients fasted for 6 h before the FDG–PET/CT scan. One hour before imaging, they received intravenous FDG (3.7 MBq/kg, 130–370 MBq). The scanning was performed from the vertex of the skull to the knee joints using an FDG–PET/CT scanner (Biograph LSO Duo; Siemens Medical Solutions, Knoxville, USA).

Two operators independently assigned FDG–PET/CT scores. FDG uptake was assessed in major joints and synovial bursae with particular attention to the shoulder, sternoclavicular, and hip joints, interspinous bursae, ischial tuberosities, and greater trochanters (these anatomical sites have been reported as PMR-specific FDG accumulation sites [8, 9, 11]). The scores were based on visual inspection and the maximum standardized uptake values (SUV_max_). In the large joints, the synovium, perisynovium, and bursae accumulate different amounts of FDG [10]. To account for this, we set up a large volume of interest (VOI) for shoulder joints to calculate the ‘total’ SUV_max_. Because of no standardized reference values for joint SUVs, we used SUV_max_ only for comparing the two groups. As a semi-quantitative scoring system on visual inspection, we used a modified Goerres et al. [7] scoring system to visually score FDG uptake: 0, no uptake (same as bone); 1, slight uptake; 2, moderate uptake (same as the liver); 3, greater uptake than the liver; and 4, uptake as strong as in the cerebellum. We considered FDG uptake scores > 2 as positive. The inter-reader agreement rate for the major joints and six PMR-specific sites was evaluated as excellent with a κ coefficient of 0.9031 (95% confidence interval (CI), 0.694–0.903). We calculated the PET-positive scores based on the cumulative number of positive accumulation sites within the interspinous bursae, ischial tuberosities, and greater trochanters, as proposed by Yamashita et al. [11].

### Statistical analysis

We analyzed between-group differences using the non-parametric Mann–Whitney U test to compare the median values of demographic data, biomarkers, and FDG–PET/CT results, and calculated 95% CIs. We considered all *p* values < 0.05 as statistically significant. We analyzed the group data using the Fisher’s exact test based on the 2012 ACR/EULAR criteria. We assessed the FDG–PET/CT predictive value to diagnose PMR based on a univariate analysis and a receiver-operating characteristic (ROC) curve analysis. We used the statistical softwares GraphPad Prism version 6 (GraphPad Software, San Diego, CA, USA) and JMP Pro 14 (SAS Institute, Cary, NC, USA) to perform all calculations.

## Results

### Patients

The study population consisted of 25 patients. The final diagnoses of the patients in the non-PMR group were malignancy (*n* = 3), infection (*n* = 2), cholesterol crystal embolism (*n* = 1), ANCA-associated vasculitis (n = 1), and unknown (n = 1). In the case of the patient with the unknown diagnosis, she did not have any detectable autoantibodies, and showed no specific findings on imaging examinations (including on the FDG–PET/CT images). She recovered within a month, taking only celecoxib. Table 1 presents the clinical characteristics of all patients, and Additional Table S1 shows the detailed clinical characteristics of patients in the non-PMR group. We found no significant differences in terms of gender, age, or inflammatory variables between the PMR and non-PMR groups. Among the 17 patients in the PMR and the eight in the non-PMR groups, 16 (94%) and 6 (75%), respectively, satisfied the 2012 ACR/EULAR provisional classification criteria for PMR. Thus, we could not differentiate the two groups using those criteria. We found elevated MMP-3 levels in both of the groups, and the mean value was significantly higher in the PMR group. In comparison with patients with RA who visited our institute during the same time, the mean MMP-3 level (168.4 ng/mL; range, 26.8–478.3 ng/mL) in the 31 patients with untreated RA was the same as that in the non-PMR group, but significantly lower than that in the PMR group (Table S2).

**Table 1:** Patient characteristics

	PMR (*n* = 17)	non-PMR (*n* = 8)	*p* value
Gender (male/female)	9/8	4/4	0.15
Age (mean ± SD years)	75 ± 2	67 ± 5	0.76
2012 ACR criteria			
Fulfills required criteria (%)	16 (94)	6 (75)	0.23
Scoring algorithm points (median (range))	4.35 (2–6)	4.13 (1–6)	0.75
ESR (median (range), mm/hour)	107 (47–160)	111 (71–141)	0.76
CRP (median (range), mg/dL)	7.9 (1.5–18.2)	8.8 (1.9–16.3)	0.69
MMP-3 (median (range), ng/mL)	421 (31.3–1074)	170 (48.5–419.5)	0.04

We measured MMP-3 levels in 14 patients with PMR and in seven patients in the non-PMR group

### FDG uptake scores at each site

**Fig. 1:**
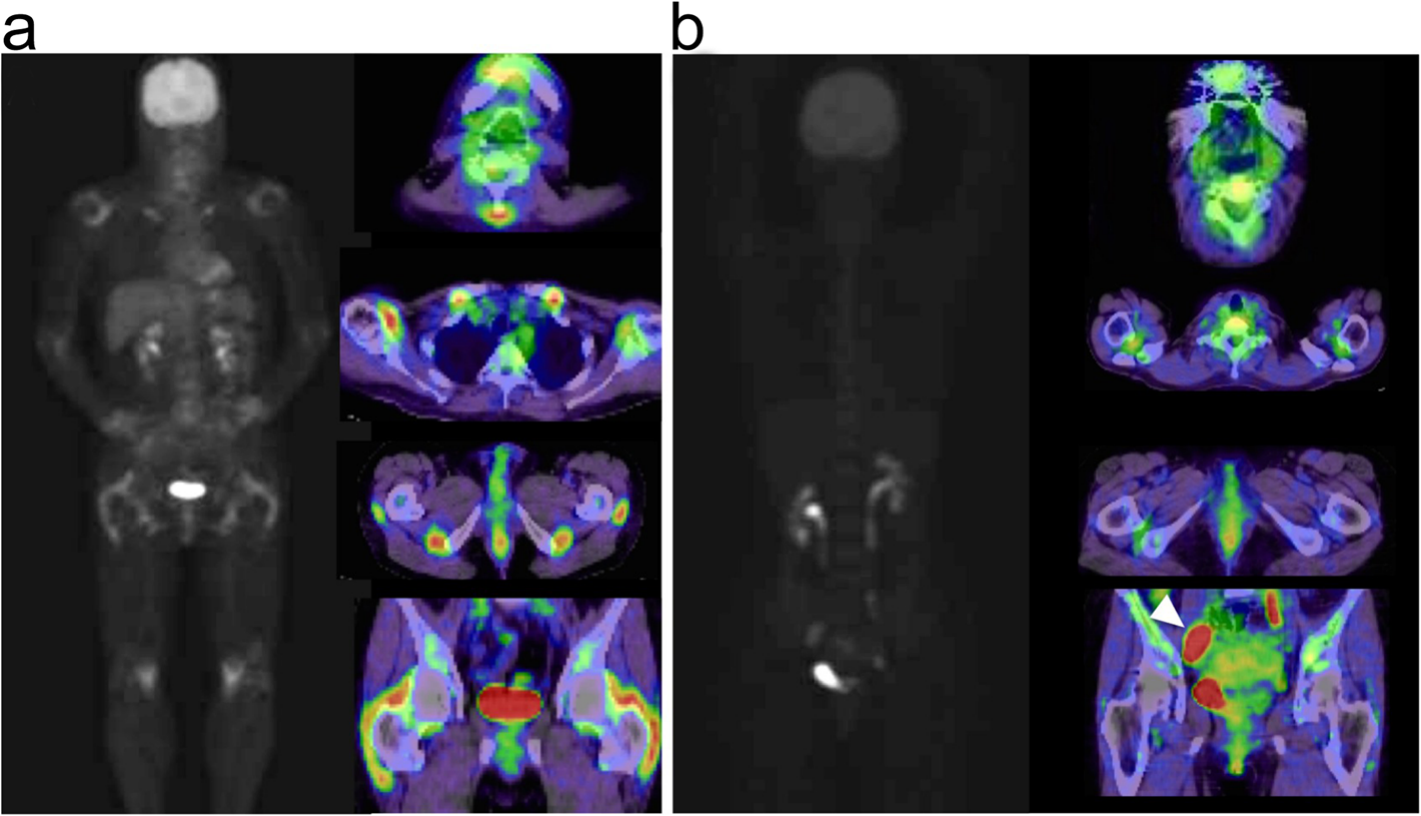
**a** Typical FDG–PET/CT findings in a patient with PMR. High FDG uptake seen in PMR-specific sites, including cervical and lumbar interspinous bursae, shoulders, sternoclavicular, and hip joints, greater trochanters, and ischial tuberosities. **b** FDG–PET/CT in a 50-year-old woman with ovarian cancer. Strong FDG uptake in the ovary (arrowhead). Weak FDG uptake in the shoulders but not in any other PMR-specific sites

Table 2 summarizes the FDG uptake scores. Similar to the results in other studies on PMR patients, we found FDG accumulation (by both uptake score and SUV_max_) in all the PMR-specific sites. Figure 1a shows the typical FDG–PET/CT findings in patients with PMR. Additionally, we found FDG accumulation in the sternoclavicular joints.

**Table 2:** FDG uptake in specific sites of patients in the PMR or non-PMR (polymyalgia-like illness) groups

FDG accumulation sites		PMR *(n* = 17)	non-PMR (*n* = 8)	*p* value (CI)
Shoulder	FDG uptake score	4.00 (0–4)	1.50 (0–3)	0.0001 (1–4)
	SUV_max_	5.25 (2.18–6.72)	2.54 (1.69–3.51)	0.0008 (0.85–3.38)
Sternoclavicular joint	FDG uptake score	2.00 (0–4)	0.50 (0–2)	0.1045 (0–2)
	SUV_max_	2.53 (1.18–4.51)	1.68 (1.27–3.19)	0.0749 (−0.23–1.54)
Interspinous bursae	FDG uptake score	3.00 (0–4)	0.00 (0–2)	0.0012 (1–4)
	SUV_max_	3.85 (1.36–14.80)	2.21 (1.22–4.05)	0.0061 (0.56–3.03)
Hip	FDG uptake score	4.00 (0–4)	0.00 (0–2)	0.0039 (1–3)
	SUV_max_	3.47 (1.13–7.10)	1.93 (1.44–2.71)	0.0073 (0.25–2.76)
Greater trochanter	FDG uptake score	2.00 (0–4)	0.50 (0–3)	0.0442 (0–3)
	SUV_max_	2.86 (1.33–4.80)	2.03 (1.48–3.00)	0.1026 (−0.12–1.83)
Ischial tuberosity	FDG uptake score	3.00 (0–4)	0.50 (0–3)	0.0183 (0–3)
	SUV_max_	3.01 (1.42–7.18)	2.76 (1.54–3.70)	0.1146 (−0.22–2.65)
PET-positive score		2.00 (0–3)	1.00 (0–3)	0.0086 (0–2)

PET-positive score: the number of FDG accumulations in interspinous bursae, ischial tuberosities, and greater trochanters

Patients in the non-PMR group had significantly lower FDG accumulation in PMR-specific sites (Fig. 1b) and showed FDG accumulation, such as the location of malignancy (Table S1). The median semi-quantitative FDG uptake scores in patients in the non-PMR group were significantly lower than those in the patients with PMR in the shoulder, interspinous bursae, and hip and ischial tuberosities (Table 2). The median PET-positive score in patients in the non-PMR group was significantly lower than in the patients in the PMR group (Table 2).

All FDG accumulations in PMR-specific sites except sternoclavicular joint were identified as predictors of PMR in our univariate analysis (Table 3). In ROC analysis, the sensitivity and specificity of FDG accumulation in the shoulder joints were higher than the others.

**Table 3:** Univariate analysis and ROC curve analysis of the FDG–PET/CT findings to diagnose PMR

FDG accumulation sites	Odds ratio	95%CI	*p* value	Sensitivity (%)	Specificity (%)
Shoulder	3.84	1.710–14.124	0.0003	88.2	75.7
Sternoclavicular joint	2.229	0.990–6.812	0.0534	52.9	40.4
Interspinous bursae	3.024	1.419–8.382	0.0025	82.4	57.4
Hip	3.213	1.547–9.715	0.0006	64.7	64.7
Greater trochanter	2.102	1.150–5.117	0.0284	64.7	39.7
Ischial tuberosity	2.215	1.075–5.188	0.0156	58.8	46.3

## Discussion

Histopathological studies have revealed that the synovium, capsule, and bursae of the shoulder of patients with PMR have inflammatory changes with infiltration of T lymphocytes and macrophages, and increased vascularity [16, 17]. Various imaging tests including US and MRI have been used to detect inflammation to diagnose PMR [8]. Adding US results increased the sensitivity and specificity (66 and 81%, respectively) over the diagnostic criteria alone; however, this increase is relatively small [15]. No US consensus for PMR diagnosis exists in terms of involved joints, range of sites to be examined, or specific findings [8, 15].

MRI is a useful technique to detect inflammatory changes in joints and adjacent tissues. Reports have described various MRI findings in PMR [8].

Similar to other published results [11] from PMR cases, we found FDG accumulation in all reported PMR-specific sites, and in sternoclavicular joints like the report using bone scintigraphy [8]. Moreover, all PMR patients in the study had PET-positive scores > 2. Three cases in the non-PMR group had FDG accumulation in one site (shoulder, ischial tuberosity, or greater trochanter); however, their PET-positive scores were < 2.

While US, MRI, and FDG–PET/CT may all detect tissue inflammation in patients with PMR, whether these three imaging examinations can discriminate between polymyalgia-like illnesses and PMR is not clear. In the 2012 ACR/EULAR provisional classification criteria for PMR, adding an US examination decreased the specificity for discriminating RA from PMR to 65% [15]. Ochi et al. reported that MRI findings in severe rotator cuff tendinopathy, periarticular soft tissue edema, and large effusions in and around the shoulder and hip joints are useful indicators for diagnosing PMR, and also for discriminating RA from PMR [18].

Takahashi et al. reported the differences in FDG–PET/CT findings between patients with PMR and those with elderly-onset RA: In the shoulders and hips, they observed specific uptake patterns in each group with circular and linear uptake patterns around the humeral head in the case of RA, and focal and non-linear uptake patterns in the case of PMR [19]. Moreover, focal uptake in front of the hip joint, indicating iliopectineal bursitis, tended to be limited to the patients with PMR [19].

We did not include RA patients in our non-PMR group because no RA patients within the study period fulfilled the Bird’s criteria, and imaging examination is an ancillary procedure for diagnosis in the routine clinical practice. When discriminating RA, physicians look for peripheral small joint arthritis, the presence of serum rheumatoid factor and anti-CCP antibody, and the diagnostic RA criteria [20]. We understand that imaging techniques like FDG–PET/CT, US, or MRI, are not the sole basis for distinguishing the different possible etiologies of inflammation in one site.

We found that FDG accumulation in the shoulder joints was a predictor with higher sensitivity and specificity for diagnosing PMR. However, a limitation of this study was that it included a small population. In addition, if we had included RA patients with shoulder joint swelling in the study, the results could have been different.

The pathogenesis of myalgia in a variety of conditions that mimic PMR is still uncertain [1]. Inflammatory changes in polymyalgia-like illnesses have not been confirmed by histopathologic examinations. In our study, patients in the non-PMR group showed significantly less FDG accumulation in PMR-specific sites than those in the PMR group. These results suggest that the PMR pathogenesis may differ from that of non-PMR illnesses, even if the clinical presentations are similar. Inflammatory changes with infiltration of lymphocytes and macrophages, and increased vascularity may not be seen in the synovium and bursae of non-PMR disorders.

In this study, the mean MMP-3 level was significantly higher in the patients in the PMR group than in those in the non-PMR group and untreated RA patients. However, we also found the level of MMP-3 to change over a wide range. Thus, the MMP-3 value may be misleading in individual cases. More data is needed to determine whether a threshold level for serum MMP-3 that can distinguish between these disorders exists or if other blood tests can be combined with MMP-3 values to diagnose PMR.

To diagnose polymyalgia-like illnesses, physical examination findings need to be carefully considered, and blood tests and imaging tests should be planned to address a wide range of diagnoses. An FDG–PET/CT may be helpful, not only for differentiating PMR from polymyalgia-like illnesses, but also for determining the correct underlying diagnosis.

## Conclusions

Various patterns of FDG uptake in patients with polymyalgia-like illnesses reflect the diversity of disorders despite the similar clinical presentations. Together with the current diagnostic criteria, the FDG accumulation pattern in PMR-specific sites may increase the accuracy of PMR diagnoses.

## Supplementary information

**Additional file 1: Table S1**. Description of data: Clinical characteristics of eight patients in the non-PMR group who fulfilled Bird’s diagnostic criteria for PMR

**Additional file 2: Table S2.** Description of data: Clinical characteristics of patients with PMR and 31 untreated patients with RA having presented the disease for less than 6 months

## Data Availability

The datasets during and/or analyzed during the current study available from the corresponding author on reasonable request.

## Abbreviations

CRP: C-reactive protein
ESR: Erythrocyte sedimentation rates
PET: Positron emission tomography/computed tomography
PMR: Polymyalgia rheumatica
ROC: Receiver-operating characteristic
VOI: Volume of interest
