# Supplementary material for: 18F-fluorodeoxyglucose–positron emission tomography/computed tomography for the diagnosis of polymyalgia-like illnesses: a retrospective study
Source: BMC Rheumatol. 2020 Apr 24;4:21. doi: 10.1186/s41927-020-00121-y (PMC7181584; doi:10.1186/s41927-020-00121-y)
Supplement: Supplementary file 1 — Additional file 1: Table S1. Description of data: Clinical characteristics of eight patients in the non-PMR group who fulfilled Bird’s diagnostic criteria for PMR [file 41927_2020_121_MOESM1_ESM.docx]

| Case | Diagnosis | Atypical symptoms or laboratory findings | FDG accumulation sites except PMR-specific sites |
| --- | --- | --- | --- |
| 1 | Ovarian cancer | Positive anti-double stranded DNA antibody | Ovary |
| 2 | Rectal cancer | Positive rheumatoid factor | Rectum |
| 3 | Rectal cancer | None | Rectum |
| 4 | Infected endometriosis cyst | High fever | Ovary |
| 5 | Cholesterol crystal embolism | Red blood cell casts in urinal sedimentation | Lymph nodes |
| 6 | Coxsackie virus infection | Skin rash | Lung |
| 7 | ANCA-associated vasculitis | Positive rheumatoid factor and MPO-ANCA | Lung |
| 8 | Unknown | None | Lymph nodes |

Table S1. Clinical characteristics of eight patients in the non-PMR group who fulfilled Bird’s diagnostic criteria for PMR
