# Supplementary material for: 18F-fluorodeoxyglucose–positron emission tomography/computed tomography for the diagnosis of polymyalgia-like illnesses: a retrospective study
Source: BMC Rheumatol. 2020 Apr 24;4:21. doi: 10.1186/s41927-020-00121-y (PMC7181584; doi:10.1186/s41927-020-00121-y)
Supplement: Supplementary file 2 — Additional file 2: Table S2. Description of data: Clinical characteristics of patients with PMR and 31 untreated patients with RA having presented the disease for less than 6 months [file 41927_2020_121_MOESM2_ESM.docx]

Table S2. Clinical characteristics of patients with PMR and 31 untreated patients with RA having presented the disease for less than 6 months

|  | PMR (n = 17) | RA (n = 31) | p value (CI) |
| --- | --- | --- | --- |
| Gender (male/female) | 9/8 | 14/17 |  |
| Age (median (range), years) | 77 (60–89) | 68 (26–82) | 0.0014 (−16 to −3) |
| ESR (median (range), mm/hour) | 107 (47–160) | 29 (8–119) | <0.0001 (−92 to −48) |
| CRP (median (range), mg/dL) | 7.9 (1.5–18.2) | 0.5 (0.3–17.9) | <0.0001 (−8.3 to −4.5) |
| MMP-3 (median (range), ng/mL) | 421 (31.3–1074) | 161.4 (26.8–478.3) | 0.04 (−346.5 to −1.1) |

MMP-3 was measured in 14 patients with PMR and in all patients with RA.
